# Climate trauma from wildfire exposure impacts cognitive decision-making

**DOI:** 10.1038/s41598-025-94672-0

**Published:** 2025-04-16

**Authors:** Jason Nan, Satish Jaiswal, Dhakshin Ramanathan, Mathew C. Withers, Jyoti Mishra

**Affiliations:** 1 Neural Engineering and Translation Labs (NEATLabs), University of California, San Diego, 9500 Gilman Drive, Mail Code 0737, La Jolla, CA 92037 USA; 2 Department of Psychiatry, University of California, San Diego, La Jolla, CA USA; 3 Department of Bioengineering, University of California, San Diego, La Jolla, CA USA; 4 Department of Mental Health, VA San Diego Medical Center, San Diego, CA USA; 5 Center of Excellence for Stress and Mental Health, VA San Diego Medical Center, San Diego, CA USA; 6 Department of Psychology, Utah Valley University, Orem, UT USA

**Keywords:** Decision-making, Reward, EEG, Alpha, Posterior cingulate cortex, Decision, Neural circuits, Reward

## Abstract

Climate trauma refers to the chronic mental health sequalae of climate disaster events. We have previously shown evidence for such trauma with accompanying anxiety and depression symptoms after California’s 2018 Camp Fire wildfire. Here, we investigate whether this climate trauma also impacts cognitive decision-making and its neural correlates. One year after the wildfire, we recruited three groups - those directly exposed (*n* = 27), indirectly exposed (community members who witnessed the wildfire but not directly exposed, *n* = 21), versus non-exposed controls (*n* = 27). Participants performed a decision-making task that led to immediate and cumulative point rewards on each trial with simultaneous electroencephalography (EEG) recordings. We evaluated Win-Stay behavior in choosing to stay with the greater expected value (cumulative reward) option. Directly-exposed individuals showed significantly reduced Win-Stay behavior relative to the other groups. EEG analyses showed significantly greater parietal alpha activity for the selected choice and ensuing rewards in directly fire-exposed individuals, with an underlying cortical source of this activity in posterior cingulate cortex. Overall, these findings suggest that climate trauma may significantly impact neuro-cognitive processing in the context of value-based decision-making, which may serve as a useful biomarker target for future mental health interventions in climate change impacted communities.

## Introduction

Our changing climate is posing a global crisis that has drawn the attention of health scientists worldwide to understand and address the impacts of extreme weather events and climate disasters^1,2^. In the western United States, warming of 1.5 °C over the last 30 years has paralleled a ~ 1000% increase in annual forest-fire area^3^. As climate change accelerated disasters such as the wildfires in the western US become frequent, significant impacts are being observed not just on human physical health but also mental health^4–6^.

Our recent studies in communities impacted by California’s deadliest wildfire to-date, the Camp Fire of 2018, have demonstrated mental health as well as neuro-cognitive impacts of wildfire exposure^7,8^. In a sample of 725 California residents, Silveira et al.^8^ found that individuals directly exposed to the wildfire disaster, showed significantly pronounced symptoms of post-traumatic stress disorder (PTSD), depression and anxiety even a year after the wildfire event. Such complex mental health impacts of a climate disaster event have been referred to as *climate trauma*^9^.

There are very few studies that have investigated the cognitive and neurobiological impacts of climate disasters^10^. In a recent empirical study, we examined a range of core cognitive abilities in individuals affected by the 2018 California wildfire disaster^7^. These cognitive assessments were conducted one year after the wildfire event and included tasks of selective attention, response inhibition, interference processing, working memory and emotion bias. This study found a significant and selective deficit in interference processing, i.e., the ability to deal with distractions in individuals who had suffered from climate trauma from the wildfire disaster relative to those who did not. Additionally, electroencephalographic (EEG) recordings conducted simultaneous to the cognitive tasks showed significantly greater activity in frontal cortex in individuals who were directly fire-exposed relative to others, and specifically for the impacted interference processing task. Notably, this state of frontal hyperarousal observed under climate trauma also dovetails with evidence for frontal cortex hyperexcitability observed in PTSD^11,12^, and may reflect the greater cognitive effort needed to process irrelevant distractions^13,14^.

One of the critical cognitive functions affected by psychological trauma, in general, is decision-making^15,16^, particularly in the form of deficits in reward functioning^17^. Studies show that individuals with PTSD exhibit higher approach-aversion conflict when making decisions^18^ and show changes in reward processing often characterized as anhedonia^17^. Furthermore, decision-making is heavily influenced by fundamental attention and distraction processes^19^, which we have already shown to be impacted by climate trauma^7^. However, findings are still mixed regarding whether and how more complex decision-making and associated rewards are affected in PTSD^17,20^. Yet, recovering from trauma of any kind, including climate trauma, requires optimal reward processing, which builds intrinsic motivation and reconnection with positive experiences and thereby, supports stress resilience^21,22^. Thus, understanding of cognitive and neural mechanisms underlying decision-making and associated reward processing can offer insights into novel diagnostic methods and personalized treatment strategies for trauma impacts.

To the best of our knowledge, the current research is a first to study cognitive and neural processes during decision-making and ensuing reward processing in the context of climate trauma. For this study, individuals who had been exposed to the 2018 California wildfire disaster as well as non-exposed controls participated in a simple, two-choice decision-making task^23^, 6–12 months after the wildfires. Study participants choose between one probabilistic choice deck that yielded small frequent gains but had low expected value (EV) of cumulative gains over time versus a second choice deck that yielded higher EV cumulative gains but had small frequent losses. We chose this two-choice task as it is able to distinguish decision-making bias towards frequent small gains versus longer-term EV-based choices, which can be confounded in other multi-choice tasks^24,25^. Specifically, we focused on assessing preference for the large EV choices as it marks the important ability for reward learning over time and foresighted long-term decision-making. We used the Win-Stay behavior metric to evaluate individual preference for large EV choices^23,26–28^; this metric quantifies the ability to stay with (i.e., continue to choose) the deck that delivers higher EV after experiencing a win (i.e., gain) on this deck. This Win-Stay metric has also been shown to be more sensitive at distinguishing behavior on choices that differ in EV than comparing the overall proportion of choices made for contrasting options^23^. It has also been shown that individuals with PTSD sacrifice rewards in the presence of negative stimuli^29^, and show deficits in reward learning^16^. Hence, it is possible that individuals exposed to climate trauma may be averse to frequent losses even if that choice ultimately has higher EV and/or fail to learn the distinction between low vs. high EV choices given impacted attention and distractor processing^7,19^. Therefore, we hypothesize that long-term decision-making ability, indexed by Win-Stay behavior on the higher EV choice, may be impacted by climate trauma in the context of a wildfire disaster.

We further hypothesize that effects of climate trauma on decision-making ability may be linked to underlying neural dynamics, especially within fronto-parietal brain regions that dictate attention and decision-making. There is convergent evidence from healthy participants as well as lesion studies demonstrating the critical role of the fronto-parietal regions in decision-making^30–33^. Relevant to the current research, in a functional neuroimaging study Paulus et al.^32^ showed that response inconsistencies on Win–Stay (or Lose–Shift) behavior in a two-choice prediction task are linked with activation of parietal cortex. Given the lack of neural evidence in the context of climate trauma, other neurophysiological studies of non-climate trauma may serve as a reference guide, and have also implicated atypical processing in fronto-parietal brain regions^21,34,35^. Thus, overall, this study hypothesizes that cognitive decision-making dynamics indexed by the Win-Stay measure may differ for individuals who have experienced climate trauma and further may be associated with altered neural dynamics in fronto-parietal brain regions.

## Methods

### Participants

 This study included 75 participants (mean age: 24.57 ± 6.20 years, range: 18–47 years, 63 females), who took part in the cognitive and neural decision-making evaluation and were a subset of participants sampled in our previous wildfire study^8^. All participants were sampled at 12 months after the 2018 Camp Fire in Northern California, i.e. all study data was collected prior to the COVID-19 pandemic period. This sample included three groups of participants: directly exposed to the wildfire (*n* = 27), indirectly exposed to the wildfire (*n* = 21), and non-exposed controls who were age and gender-matched to the directly exposed group (*n* = 27). The groups were classified based on self-reports on the Life Events Checklist 5 ^8^, i.e., in the context of the fire, the three groups responded as ‘happened to me personally’ for the directly exposed group; ‘witnessed it happen to someone else’ for the indirectly exposed group; and ‘learned about it or not applicable’ for non-exposed controls, respectively. An additional group was created called ‘other’ which comprises of the indirectly exposed and non-exposed control groups.

All participants provided written informed consent for the study approved by the local university Institutional Review Board (IRB) and in accordance with the Declaration of Helsinki. Specifically, the directly and indirectly exposed participants were located at California State University (CSU) at Chico, within 10–15 miles of the Camp Fire, and were approved by the CSU Chico IRB#22838, while non-exposed controls were located in the San Diego region, 600 miles away from the Camp Fire, and were approved by the University of California, San Diego IRB#180140. The majority of participants (95%) were right-handed. All participants had normal/corrected-to-normal vision and hearing, and no participant reported color blindness. All participants had at least a high-school education.

### Demographics

 All participants provided demographic information by self-report including age, gender, and ethnicity. Socio-economic status was measured on the Family Affluence Scale^36^; this scale measures individual wealth based on ownership of objects of value (e.g., car/computer) and produces a composite score ranging from 0 (low affluence) to 9 (high affluence).

### Mental health 

All participants self-reported whether they had experienced recent trauma as per the standard PTSD checklist screen (“were you recently bothered by a past experience that caused you to believe you would be injured or killed?” 1: Not bothered at all, 2: Bothered a little, 3: Bothered a lot)^37^. Participants rated anxiety symptoms on the Generalized Anxiety Disorder: GAD7 scale^38^ and depression symptoms on the Patient Health Questionnaire: PHQ9 scale^39^.

All participant demographics and mental health characteristics have been tabulated and discussed in our previous study in which the same sample underwent other neuro-cognitive assessments^7^, and are also shown in Results Table 1.

### Experimental task

 We investigated a two-choice decision-making task^23^ that we refer to as *Lucky Door* in which participants were given the below instruction:

“You will see two doors.

Tap left or right to choose a door.

You will gain or lose coins at each door. Choose the lucky door.”

In this task, participants chose between one of two doors, either a rare gain door (RareG, probability for gains *P* = 0.3, for losses *P* = 0.7) or a rare loss door (RareL, probability for losses *P* = 0.3, for gains *P* = 0.7). Participants used the left and right arrow keys on the keyboard to make their door choice. Door choice was monitored throughout the task. The task choice decisions on each trial were response-constrained, not time-constrained, i.e. participants could take their time to select their choice.

The task consisted of two blocks, an experimental block and a baseline block that were counterbalanced across participants. In the experimental block, expected value (EV) was greater for the RareG door (*P* = 0.3 for + 60 coins, *P* = 0.7 for − 20 coins, EV = + 40) than for the RareL door (*P* = 0.3 for − 60 coins, *P* = 0.7 for + 20 coins; EV = − 40). Manipulation of EV, with greater expected value tied to the RareG door, allowed for investigating individual propensities to prioritize long-term (or cumulative) versus short-term (or immediate) rewards. The RareG door was assigned greater EV because selecting this door suggests EV magnitude-based decision processing in subjects as opposed to simply choosing based on frequency of gains, in which case the RareL choice should be preferred.

In the baseline block, EV was the same for both RareG (*P* = 0.3 for + 70 coins, *P* = 0.7 for − 30 coins, EV = 0) and for the RareL door (*P* = 0.3 for − 70 coins, *P* = 0.7 for + 30 coins; EV = 0), and allowed investigation of gain frequency bias towards the RareL door without EV differences.

**Fig. 1:**
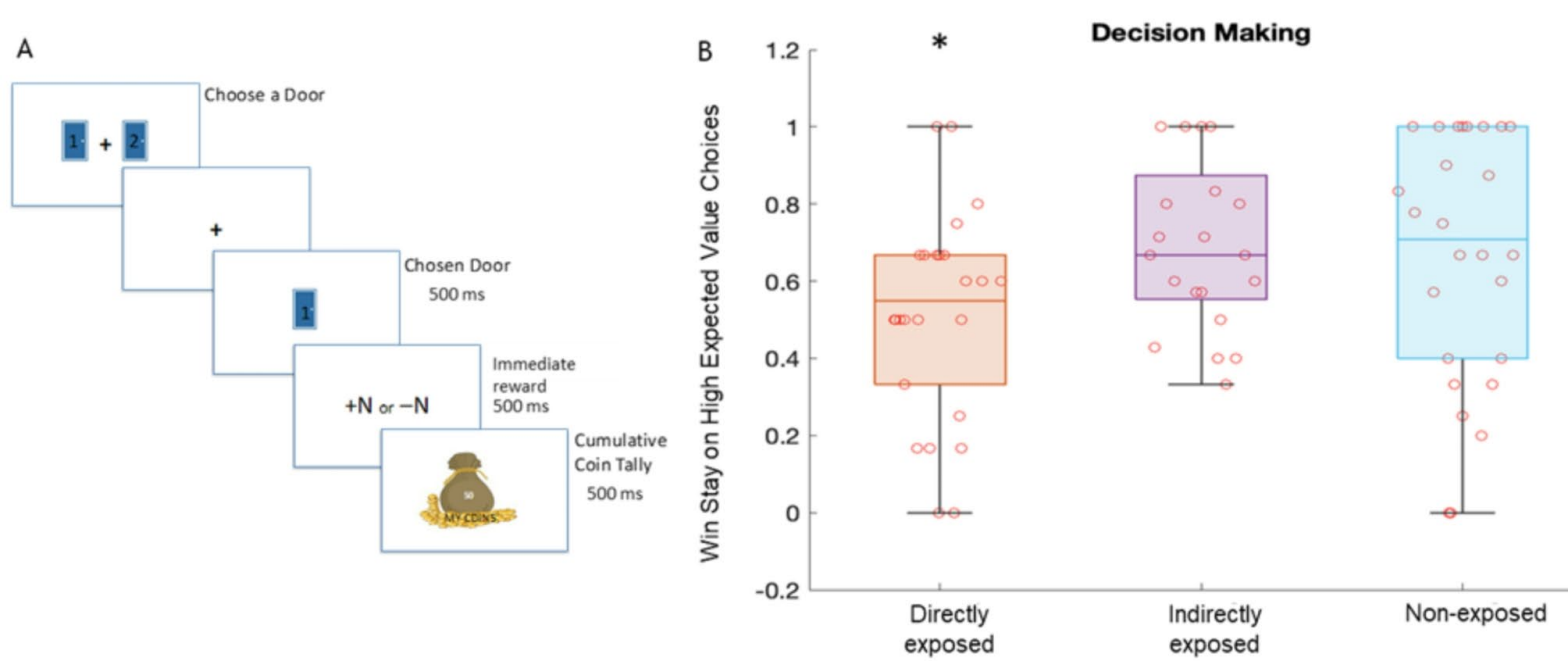
Task Design and Performance. (**A**) Flow of a *Lucky Door* task trial. On each trial, participants are initially presented with a choice of two doors. After they make a choice, they are shown the chosen door (for a duration for 500 ms), then presented with the amount of coins they received for that choice (immediate reward, shown for a duration of 500 ms) followed by presentation of their total coin tally (cumulative reward, also shown for 500 ms). (**B**) Box plot of the Win-Stay behavioral metric for high expected value (EV) choices in the experimental block of the *Lucky Door* task, showing group based probability of participants staying with the high EV door after winning coins. Individuals directly exposed to fires showed significantly lower Win-Stay behavior relative to other, i.e., indirectly exposed and non-fire-exposed study participants.

40 trials were presented per block approximating similar trial numbers as previous human reward task studies^40,41^. Figure 1A shows a schematic of the task stimulus sequence. On each task trial, a fixation cue was followed by two door choices that remained on the screen until a choice was made. After choice selection, central fixation was presented for 500-ms duration followed by selected choice presentation for 500-ms duration, then immediate reward presentation for 500-ms duration corresponding to the reward for the selected door on that trial, and then cumulative reward presentation for 500-ms duration corresponding to total reward earned until that trial during the block.

The *Lucky Door* task was deployed in Unity as part of the assessment suite on the *v2019.1 BrainE* (short for Brain Engagement) platform https://play.google.com/store/apps/details?id=com.neatlabs.braine&hl=en_US^42^. The Lab Streaming Layer (LSL^43^), protocol was used to time-stamp each stimulus/response event during the task. Study participants engaged with the assessment on a Windows 10 laptop sitting at a comfortable viewing distance.

### Behavior analysis

 Behavioral data were obtained from 74 of 75 participants, except for missing data from one participant in the control group. The main behavior metric was Win-Stay, i.e., participant’s willingness to stay with the RareG door that had greater EV (but lesser immediate gains) after they encountered a winning trial for this choice in the experimental block. Win-Stay was calculated as the ratio of times a participant stayed with the RareG choice after a win compared to total number of trials after a win. On the baseline block that had no EV differences, we also calculated Win-Stay for RareG choices as a control to confirm the hypothesis that Win-Stay behavior selectively shows group differences on the experimental block that had EV differences between choices^23^.

To analyze group differences while accounting for all covariates of age, gender, ethnicity, socioeconomic score, and mental health scores of anxiety and depression, we modeled the behavior metrics across all three groups with a linear model using the fitlm function in MATLAB with robust regression option applied to reduce outlier influence^44^.

### Sample size and power

 Our total sample size was a priori adequately powered to detect a medium effect size relationship between Win-Stay behavior and group differences in the above behavioral regression analysis at beta of 0.8 and alpha significance level of 0.05 as calculated using v3.1.9.4 of G*Power software https://www.psychologie.hhu.de/arbeitsgruppen/allgemeine-psychologie-und-arbeitspsychologie/gpower^45^. Standardized regression coefficients > 0.1 are considered small effect size, > 0.3 medium and > 0.5 are large^46^. For all other analyses, we also report effect sizes, where medium effect sizes (Cohen’s d > 0.5) can be considered scientifically meaningful.

### EEG processing

 EEG simultaneous to the decision-making task was acquired in most participants (*n* = 57) with missing EEG due to technical issues in 3 participants in the control group, 7 participants in the indirectly exposed group, and 8 participants in the directly exposed group. RareG trials were analyzed coinciding with the behavioral analyses on these trials. Since we are analyzing neural correlates related to decision making and ensuing reward processing, we segmented the trial structure into three distinct time period associated with choice defined as 0-500 ms after the chosen door is presented, immediate reward defined as 500–1000 ms after the chosen door appears, and cumulative reward defined as 1000–1500 ms after the chosen door appears. Each of the time periods are 500 ms to align with the duration of each stimulus (i.e., chosen door, immediate reward and cumulative reward) appearing on the screen. These three timings are also shown in Fig. 1A.

Neural data analyses were conducted using a uniform two-step processing pipeline published in several of our studies^7,23,42,47–54^. Step (1) EEG channel data processing was conducted using the EEGLAB toolbox v2020 in MATLAB v2022b. EEG data was resampled at 250 Hz and filtered in the 1–45 Hz range to exclude ultraslow DC drifts at < 1 Hz and high-frequency noise produced by muscle movements and external electrical sources at > 45 Hz.

There were no missing channels in the EEG data across subjects. Epoched data were cleaned using the autorej function in EEGLAB to remove noisy trials, i.e. >5SD outliers rejected over max 8 iterations, followed by further cleaning of electrooculographic, electromyographic or non-brain source artifacts using the Sparse Bayesian learning (SBL) algorithm (https://github.com/aojeda/PEB)^52^. In addition to the automatic rejection, we also implemented an amplitude criterion where any trial exceeding 100 uV was considered noisy and removed. The cleaned data were then band filtered in the physiologically relevant theta (4–8 Hz), alpha (8–13 Hz), and beta (13–30 Hz) frequency bands. Gamma band (30–70 Hz) was excluded from analysis because it requires an electronically shielded acquisition environment and more sensitive recording devices, which were not accessible for this study. Epoched events were then extracted and averaged across trials to remove single trial noise.

Step (2) We used the block-Sparse Bayesian learning (BSBL-2 S) algorithm to localize frequency band filtered EEG data and partitioned the signals into cortical regions of interest (ROIs) and artifact sources^52,55^. For the source space activations, ROIs were based on the standard 68 brain region Desikan-Killiany atlas^56^ using the Colin-27 head model^57^. BSBL-2 S is a two-step algorithm in which the first-step is equivalent to low-resolution electromagnetic tomography (LORETA^58^). LORETA estimates sources subject to smoothness constraints, i.e. nearby sources tend to be co-activated, which may produce source estimates with a high number of false positives that are not biologically plausible. To guard against this, BSBL-2 S applies sparsity constraints in the second step wherein blocks of irrelevant sources are pruned. Notably, this data-driven sparsity constraint reduces the effective number of sources considered at any given time as a solution. The sparsity is imposed at the level of cortical ROIs, thereby projecting the data onto this space of few ROIs and reducing the uncertainty of the inverse solution. Thus, it is not that only higher channel density data can yield source solutions, the ill-posed inverse problem can also be solved by imposing more aggressive constraints on the solution to converge on the source model at lower channel densities, as also supported by prior research^59,60^. Of note, the BSBL-2 S two-stage algorithm has been benchmarked to produce evidence-optimized inverse source models at 0.95AUC relative to the ground truth, while without the second stage < 0.9AUC is obtained, verified using both data and simulations^52,55^. We have also shown that cortical source mapping with this method has high test-retest reliability (Cronbach’s alpha = 0.77, *p* < 0.0001) obtained with recordings conducted one-week apart^42^.

### Neural data analysis

 Here, we applied a standardized pipeline with modifiable parameters to streamline both scalp and source space neural analyses. A github with the source code can be found in (https://github.com/jasonnan2/Automated-Analysis-of-EEG/)^61^.

This standardized pipeline included.

1. Outlier rejection on the final trial-averaged scalp and source data, which sets any datapoint > 5SD across all subjects to NaN.
2. Baseline correction was done on both scalp and source activity relative to the − 250 ms to − 50 ms fixation time window prior to choice presentation in each scalp electrode/ source ROI within each subject. This baseline was chosen as it provides a silent period of neural activity wherein no stimulus-evoked processing occurs^62^.
3. Differential scalp topography maps comparing groups were plotted for each of the three frequency bands (theta, alpha, beta) and three trial periods (choice, immediate reward, cumulative reward) for a total of 9 scalp maps. Patterns of significantly different electrodes between groups of interest were validated with permutation clustering across 10,000 iterations, and false discovery rate (FDR) corrections were applied for 9 topographic map comparisons (3 frequency bands × 3 trial periods)^63^.
4. Alpha band event-related activity was also averaged over a standard posterior alpha electrode cluster (Pz, P3, P4, and POz) for significance testing between groups. Theta and beta band average electrode clusters were not defined as activity in these bands did not differ in step 3 above.
5. To find relationships between behavior and neurophysiology, we fit linear models to test for group x neural interaction predicting behavior data (i.e., Win-Stay for large EV RareG choices). These models controlled for relevant demographic covariates. Here, the neural variable refers to average alpha activity in the posterior electrode cluster as well as in the individual component electrodes (P3, Pz, P4, and POz). Relevant demographic covariates were determined by the behavior analysis done prior to neural analysis. All models were fit with FDR corrections applied for multiple comparisons. All continuous variables were z score standardized for the models so effect size can be reported as standardize beta values.
6. Withing-group Spearman’s correlations were used to follow-up on any significant neuro-behavioral group interactions obtained in step 5 above.
7. We conducted cortical source localization analysis for any relevant scalp electrode activities that showed significant group differences and neurobehavioral correlations (i.e., alpha activity in the 500 ms choice period per the Results).

## Results

### Behavioral performance

 The two-choice decision-making task design and corresponding Win-Stay behavior performance on high EV (i.e., rare gain or RareG) choices that resulted in greater long-term cumulative reward, are shown in Fig. 1. We implemented a robust linear regression of Win-Stay behavior with participant group as predictor and also included covariates of age, gender, ethnicity, socioeconomic scores, anxiety and depression shown in Table 1. The regression model was overall significant (adjusted *R* = 0.35, Fstat = 2.38, *p* = 0.03), and notably, showed a significant effect only for the directly-exposed group (standardize β=-0.76±0.34, tstat=-2.2, *p* = 0.03) but not for the indirectly-exposed or non-exposed group (*p* > 0.57). Thus, only the directly-exposed group showed lower Win-Stay choices relative to the other two groups. Also, age was the only significant covariate in the model (standardize β=-0.35±0.13, tstat=-2.8, *p* = 0.007).

We further modeled gain frequency bias based on group and including all demographic and mental health covariates per Table 1. But this model was not significant (adjusted *R* = 0.23; Fstat = 1.52, *p* > 0.1). Also, Win-Stay behavior on RareG trials on the baseline block that had no EV differences between choices, did not have a significant regression model (adjusted *R* = 0.31, Fstat = 1.96, *p* = 0.07) and with no effect of group (*p* > 0.1). Thus, we confirmed our hypothesis that Win-Stay behavior only differs when there are EV differences between choices.

### Neural processing

As there were no significant behavioral differences between the indirectly exposed group and non-exposed groups, for neural analyses we combined these into one group (*Other*) to compare against the directly exposed group. Summary of demographic characteristics & self-reported mental health for all groups are shown in Table 1.

**Table 1:** Demographic characteristics and self-reported mental health for participants by group.

Demographics and mental health	Directly exposed (*n* = 27)	Indirectly exposed (*n* = 21)	Not exposed (*n* = 27)	Other (*n* = 48)
	Mean ± STD	Mean ± STD	Mean ± STD	Mean ± STD
Age	24.4 ± 5.9	25.7 ± 7.0	23.9 ± 5.9	24.6±6.4
Gender n (%)				
Male	4 (14.8)	4 (19.0)	4 (14.8)	8 (16.7)
Female	23 (85.2)	17 (81.0)	23 (85.2)	40 (83.3)
Ethnicity n (%)				
Caucasian	21 (77.8)	12 (57.1)	8 (29.6)	20 (41.7)
Black/African American	1 (3.7)	0 (0)	0 (0)	0 (0)
Asian	0 (0)	2 (9.5)	11 (40.7)	13 (27.1)
More than one ethnicity	4 (14.8)	5 (23.8)	6 (22.2)	11 (22.9)
Other	1 (3.7)	2 (9.5)	2 (7.4)	4 (8.3)***
SES	4.0 ± 1.7	4.0 ± 1.7	4.9 ± 2.0	4.5±1.9
Recent trauma N (%)	18 (66.7)	3 (14.3)	0 (0)	3 (6.3)***
Anxiety (GAD7)	10.1 ± 6.6	9.7 ± 5.2	3.2 ± 2.1	6.0±5**
Depression (PHQ9)	8.9 ± 6.5	11.8 ± 6.1	2.6 ± 2.1	6.6±6.3

Stars indicate significant differences between the directly exposed and other groups. *P*-values are from non-parametric rank sum test comparisons between groups for all variables except gender and ethnicity for which Χ^2^ tests were used. (*** *p* < 0.001; ** *p* < 0.01).

**Fig. 2:**
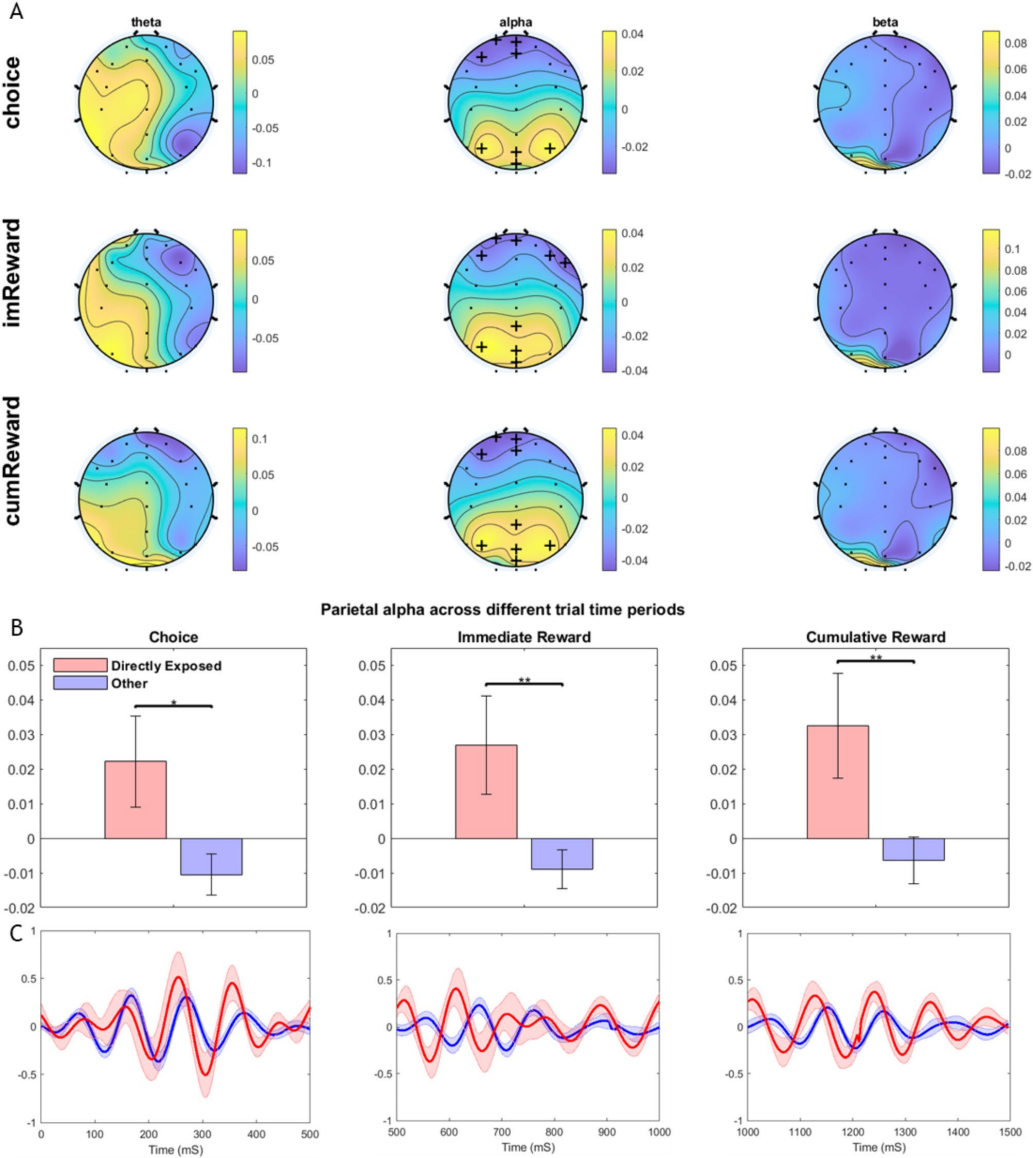
Neural activity contrasted in directly exposed vs. other (i.e., indirectly exposed and non-exposed combined) groups. (**A**) Baseline corrected scalp topography plots are shown for the 500 ms choice period, immediate reward period and cumulative reward period in theta, alpha and beta frequency bands contrasting activity in the directly exposed vs. other group participants. ‘+’ points represent permutation corrected significant electrode clusters at *p* < 0.0001. (**B**) Bar graphs show activity in the parietal alpha cluster (P3, Pz, P4, POz) observed to be significantly greater in the directly exposed (red) vs. other group (blue). Activity values are in µV. **: *p* < 0.01, *:*p* < 0.05 (**C**) Time course of event-related alpha band activity is shown at electrode Pz for the directly exposed (red) vs. other group (blue). Shaded boundaries represent standard error of the mean.

Figure 2A shows EEG scalp topographies contrasting group neural activity in the directly exposed vs. other group in theta, alpha and beta frequency bands within the 500 ms time period after choice, immediate reward and cumulative reward presentations. Electrodes showing significant group differences (i.e., directly exposed vs. other) after permutation clustering are marked with + (*p* < 0.0001), and notably appeared only in the alpha band. Given the known posterior parieto-occipital origins of alpha band activity^64–71^ and its typical topography appearing in our scalp maps (Fig. 2A), we further quantified parietal cluster alpha (at Pz, P3, P4, and POz electrodes) in grouped bar graphs in Fig. 2B. Parietal alpha differences were consistently found during the choice (effect size, Cohen’s d = 0.72; t(54)=-2.6; *p* < 0.05), immediate reward (Cohen’s d = 0.78; t(54)=-2.8; *p* < 0.01) and cumulative reward periods (Cohen’s d = 0.75; t(54)=-2.7; *p* < 0.01) as compared in between-group t-tests. We also checked that parietal alpha activity did not significantly differ between the indirectly exposed and non-exposed groups that were combined in the other group (*p* > 0.43). The average time course ERP for each of the bar graphs in Fig. 2B are shown in Fig. 2C.

To investigate whether alpha activity is a neural correlate of behavior, we implemented robust regression models that predicted Win-Stay behavior on the high EV choice; predictors included group (directly-exposed vs. other), parietal alpha activity and the interaction between group and alpha activity. Since age was a significant predictor of Win-Stay behavior, it was entered as a covariate in all models. Since ethnicity and anxiety were also significantly different between the two groups (Table 1), these were also added as model covariates. No models using average alpha activity in the parietal electrode cluster (comprised of P3, Pz, P4, POz) showed a significant neural effect on behavior. Hence, we explored models for individual electrodes in the cluster, correcting for multiple comparisons across four electrodes and three time windows (choice, immediate reward and cumulative reward). In this case, only the model for Pz alpha activity during the choice period showed a significant alpha activity by group interaction (standardize β = 0.57 ± 0.27, tstat = 2.1, *p* = 0.04). The overall model was significant (adjusted *R* = 0.46, F-stat = 3.34, *p* < 0.007), and also showed a significant effect of group (standardize β = 0.62 ±0.3, tstat = 2.0, *p* = 0.048) and age (standardize β= -0.42 ± 0.13, tstat=-3.2, *p* = 0.002). Anxiety (*p* > 0.1) and ethnicity (*p* > 0.4) were not significant covariates, and there was also no effect of alpha activity alone (*p* > 0.3). No significant neural effects were observed in the immediate/cumulative reward periods at any of the parietal electrodes.

**Fig. 3:**
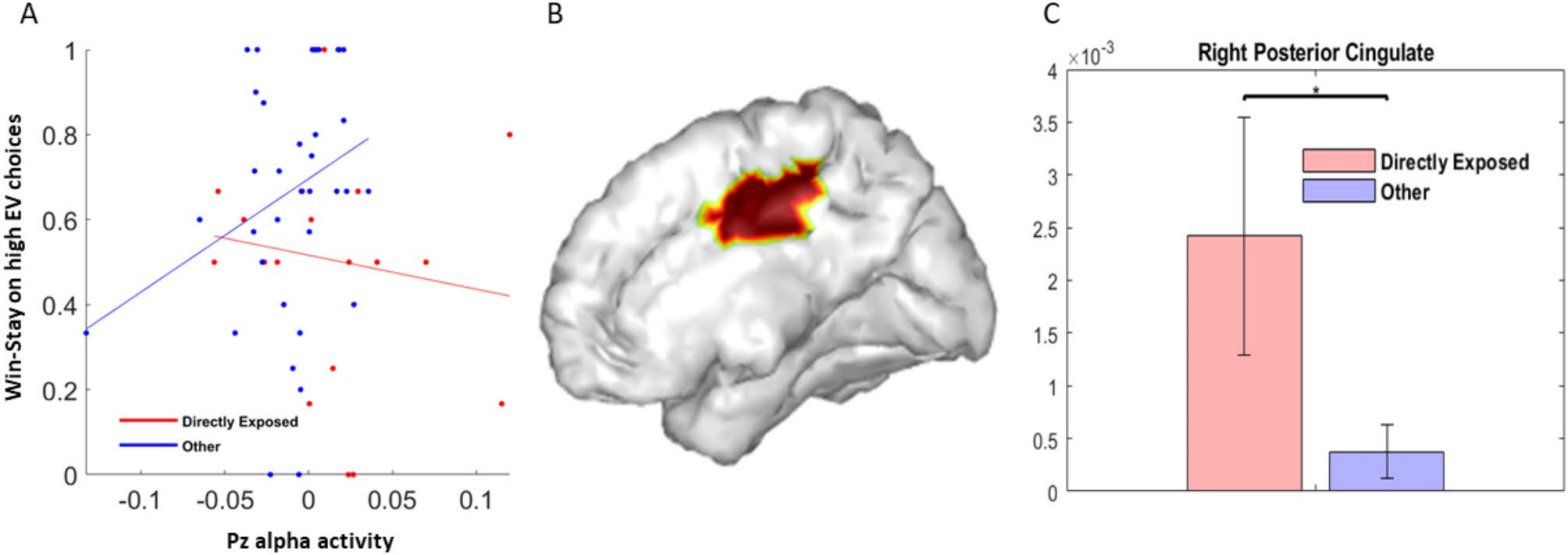
Neurobehavioral model relating posterior alpha activity to win-stay behavior (**A**) Pz alpha activity during the choice period showed a differential relationship with Win-Stay behavior within each group. Within-group Spearman’s correlations show a positive neurobehavioral correlation in the other group (*r* = 0.34 *p* = 0.04), but not in the directly exposed group (*p* = 0.5). (**B**) Alpha activity during the choice period localized to a cortical source in the posterior cingulate cortex (PCC) region. (**C**) Right PCC alpha activity was greater in the directly exposed vs. other group (*p* < 0.05, arbitrary source units).

Figure 3A illustrates the group specific alpha activity response at electrode Pz as it relates to Win-Stay behavior; a significant Spearman’s correlation was observed only in the other group (rho = 0.34 *p* = 0.04) but not in the directly exposed group (*p* = 0.5). Figure 3B shows the cortical source localization of the Pz alpha activity during the choice period masked by significant difference between activity in the directly exposed vs. other group; the source region as highlighted in the figure was observed to be right posterior cingulate cortex with greater activity in the directly exposed than the other group (Cohen’s d = 0.65; t(54)=-2.33; *p* < 0.05) as seen in Fig. 3C.

## Discussion

In the current study, our main objective was to investigate how mental health trauma from a major wildfire disaster may affect cognitive decision-making of impacted community members. For this, we specifically investigated the ability to engage and stay with high expected value choices, marked by the Win-Stay behavior metric obtained on high EV trials. We further investigated the neural dynamics of such behavioral modulation as affected by direct wildfire exposure. We observed that individuals directly exposed to the climate trauma event showed significantly lower ability to stay with the high EV choice after winning compared to indirectly exposed community participants (who witnessed the wildfire in their community but were not directly impacted) and non-exposed control participants. Additionally, there were no behavioral differences in Win-Stay behavior between the indirectly exposed and non-exposed participants. At the neural level, across three pertinent processing time windows of selected choice presentation, immediate reward and cumulative reward presentation, we observed significantly greater alpha band EEG activity especially over parieto-occipital brain regions in the directly exposed group vs. all other participants. Finally, a robust regression model investigating neurobehavioral relationships showed that alpha activity at the midline parietal electrode (Pz) during choice presentation could predict Win-Stay behavior with a significant alpha activity by group interaction. Together, these findings illustrate the impact of a traumatic climate event such as the deadly wildfire in California that we focused on here, on behavioral and neural correlates of decision-making.

The observation of impacted cognitive decision-making after climate trauma exposure, as indexed by the Win-Stay metric was consistent with our primary hypothesis. We hypothesized this impact based on prior studies of decision making in general, i.e., in non-climate related contexts in PTSD and depression^15–17,72,73^. For instance, Sailer et al.^16^ examined reward processing in clinically diagnosed PTSD patients using a decision-making task (adapted from^74,75^), and observed that individuals with PTSD showed lower accuracy in late phase reward learning relative to control subjects, suggesting lower efficiency of reward-based decision-making in PTSD. Similarly in the decision-making reward task we deploy here, high EV choices also need to be implicitly learned and differentiated from low EV choices, and failure of such learning would result in low Win-Stay behavior on high EV trials.

In the EEG neural recordings, we observed significantly greater parietal alpha on high EV trials in the directly-exposed group vs. all other participants. This observation is in line with previous reports in PTSD, showing involvement of fronto-parietal regions in decision-reward processes^76,77^. For example in a functional neuroimaging study on combat veterans, Howlett et al.^77^ observed an exaggerated neural response, specifically in the parietal region to surprising errors while participants were performing a probabilistic learning task. Interrogating neurobehavioral correlations, we found that parietal alpha, specifically at the Pz electrode during choice presentation showed group-specific modulations in the context of Win-Stay behavior. In the non-directly exposed (i.e., other group) participants, greater Pz alpha was associated with greater Win-Stay performance. In contrast, in the directly-exposed group, Pz alpha was generally of greater magnitude in the group as a whole but did not show modulation with Win-Stay behavior. Flexible alpha modulation during decision-making behavior has been associated with greater task-related cognitive effort in healthy participants^78,79^, which may explain our findings in the other group i.e., greater Win-Stay behavior is achieved with greater cognitive effort. Studies also suggest that reward-related learning during decision-making harnesses working memory processes^80,81^, and relatedly, prior work has shown that parietal alpha indexes working memory performance^82–84^. Thus, parietal alpha modulation in the other group participants may also suggest that they successfully recruit working memory processes for learning the high EV choices and thereby, generate greater Win-Stay performance. Overall higher parietal alpha magnitudes in the directly-exposed group may suggest greater cognitive effort, hyperarousal or even altered attention allocation in this group, but an inability to translate this to superior behavior performance. Indeed, attention is critical to decision making^19^, and greater parietal alpha in the directly exposed group in all three interrogated time periods of choice presentation as well as immediate and cumulative reward presentation may be reflective of hypervigilant attentive processing.

The between-group difference in parietal alpha activity in scalp EEG localized to a significant cortical source difference observed in posterior cingulate cortex (PCC), with greater activity observed in the directly exposed vs. other group. Several studies have reported the role of the PCC, a key node of the posterior default mode network, in modulation of ruminative behavior^85–88^. Rumination is also one of the primary ways in which emotion regulation is impacted in affective disorders^89,90^, and further predicts PTSD^91^. Thus, it may be plausible that directly exposed individuals under the duress of climate trauma, engage in distracted rumination behavior indexed by PCC source activity, which may affect their decision-making strategy and hence reduce Win-Stay performance.

The study’s limitations include the potential for observed group differences to be inherent traits predating the traumatic wildfire event. This constraint is common to all disaster research, as investigations typically occur post-event. Additionally, this is a first study exploring how decision-making on a simple two-choice task can be affected in populations who have suffered from climate trauma. We hypothesized that after suffering from losses in a major disaster, affected individuals may focus more on immediate small gains than stick with higher EV choices that result in cumulative gains yet have immediate small losses, i.e., these individuals may become loss averse. This hypothesis was born out in our behavioral findings as reflected by Win-Stay behavior on the higher EV choices. However, we are limited at explaining the mechanisms of these findings – do these changes reflect altered attention and greater distractibility as per our prior research^7^ and/or specific changes in reward learning and loss-aversion. Future research with more resolved neuroimaging techniques can provide more insights into mechanistic details. Further, as climate disasters become more frequent and more severe, it would be important to extend this neuro-cognitive research longitudinally to understand pre vs. post-disaster effects as well as impacts of repeated exposure, which is now unfortunately common occurrence for many of these vulnerable communities.

Among other study limitations, it has also been well-documented that individuals in lower socioeconomic strata are more vulnerable to suffering from climate related disasters^92^. However, our cohort did not have significant group differences in socioeconomic scores, hence, we cannot determine interactions between the decision-making results and socioeconomic status. Also a technical constraint is our utilization of a moderate channel density EEG system for neural recordings, and future validation could be achieved through the use of a high-density EEG or alternative neuroimaging techniques such as functional magnetic resonance imaging. Yet, notably, it is important to highlight that the choice of the moderate channel density EEG was motivated by its cost-effectiveness and feasibility within a community research setting^7^. Indeed, in such community studies, there is a crucial need to strike a balance between accessibility, feasibility, cost considerations, and data resolution^93^. We have further shown that results obtained with a moderate channel density EEG system such as the one used in this study are highly correlated to results obtained with higher density EEG systems^88^. Hence, the benefits of using greater resolution neuroimaging in future community studies should be carefully evaluated alongside cost considerations and whether those additional costs could be alternatively allocated to community service within the project scope. Future community research should also focus on procuring larger sample sizes of the neuro-cognitive data.

Overall, the current research is a first in terms of examining the effect of climate trauma on decision making. We observed that directly fire-exposed individuals showed impacted decision-making indexed by reduction in Win-Stay performance on high EV choices alongside higher alpha activity in posterior parietal regions compared to other, indirectly exposed or non-exposed study participants. Cortical source localization revealed significantly greater PCC activity in the directly exposed group suggesting that distracted rumination that often originates from PCC may be a potential contributor to impacted decision-making in this group. Future neuro-cognitively targeted trauma interventions in this context may thus aim to reduce PCC related default mode network activity. Our related intervention research with a scalable digital mindfulness and compassion training has shown significant default mode network suppression alongside enhancement of mindfulness and compassion relevant behaviors^49^. Thus, such scalable digital mental health strategies may also be tailored as potential interventions for climate trauma within impacted communities. This is especially pertinent since our prior observational studies point to mindfulness as a protective trait in this traumatic setting^8,94^. We expect to observe improved decision making post-completion of such interventions, and early access to such interventions may further prevent longer-term impacts. Indeed, mobilizing early community access to such post-disaster intervention resources is a top priority of our California wide Climate Resilience Initiative^95^.

With the planet experiencing escalating temperatures, an increasing number of individuals confront extreme climate events, and it is very important to understand impacts on cognitive health that can have future repercussions. Here, we demonstrate evidence for significantly altered neuro-cognitive processing underlying decision-making in the aftermath of a climate change accelerated wildfire event, notably observed even 12 months post-disaster. Impulsive decision-making has been shown to predict future substance use problems^96,97^. Additionally, impulsive buying has been observed as a coping strategy in the aftermath of a natural disaster^98^. This implies that impairments in cognitive decision-making may concerningly reduce the ability of individuals and communities to adapt and/or reduce investments in future-based solutions in favor of impulsive choices. These findings underscore the urgency to explore novel resiliency tools from diverse disciplines to renormalize cognitive decision-making processes immediately post-disaster to mitigate long-term impacts. The objective neuro-cognitive markers of decision-making we find here can potentially be used to guide interventions, and map the success of such intervention within climate vulnerable communities.

## Data Availability

De-identified and processed study data are available upon request from the corresponding author.
